# QAMQOR: a benchmark dataset for engagement recognition in robot-assisted autism therapy

**DOI:** 10.3389/frobt.2026.1868211

**Published:** 2026-10-13

**Authors:** Nazerke Rakhymbayeva, Aida Tungatarova, Zhansaule Telisheva, Anara Sandygulova

**Affiliations:** 1 Department of Science and Innovation, Astana IT University, Astana, Kazakhstan; 2 School of Computing and AI, Nazarbayev University, Astana, Kazakhstan

**Keywords:** children with autism, child-robot interaction, dataset evaluation, engagement recognition, machine learning, robot-assisted therapy

## Abstract

Reliable engagement recognition is important for adaptive robot-assisted autism therapy (RAAT). However, progress remains constrained by the scarcity of publicly available benchmarks evaluated under realistic deployment conditions. Existing studies commonly adopt random train–test splits, which can inflate reported performance because recordings from the same child or therapy session may appear in both the training and test sets. To address this gap, we introduce QAMQOR, a benchmark dataset for engagement recognition collected during RAAT sessions with 34 children with Autism Spectrum Disorder (ASD), aged 3−12 years. The dataset includes visual engagement annotations derived from therapy session videos and feature representations extracted using two pose-estimation frameworks. The QAMQOR dataset defines four clinically relevant evaluation protocols based on Random, Child-Independent, Session-Independent, and Activity-Independent splits. These evaluation protocols assess model generalization to unseen children, therapy sessions and activity tasks. We benchmark classical machine learning classifiers together with temporal and sequence-learning baselines for both binary and multi-class engagement recognition. Experimental results show that the evaluation protocol influenced reported performance to a degree comparable to, and for fine-grained engagement classification exceeding, the influence of classifier selection: mean macro-F1 varied by 0.12 across the four protocols in the multi-class task, against 0.07 across discriminative classifiers. The Child-Independent split proved substantially more challenging than the Random split. Under the same conditions (windows, protocols, training schedule and untuned hyperparameters) a Temporal Convolutional Network (TCN) did not outperform the classical baselines. Applying identical imbalance handling to every model improved all imbalance-sensitive metrics but left this ordering unchanged, indicating that the result is not an artifact of missing class weights. Across the models evaluated, reported performance was shifted more by evaluation design and by imbalance handling than by architectural choice. Therefore, QAMQOR serves as a reproducible reference point for this task. It also shows why fair evaluation matters: keeping the same child or session out of both the training and test sets is needed to compare future methods on equal terms and to build engagement models that transfer to new children, sessions and activities in RAAT systems.

## 1 Introduction

Engaging children with Autism Spectrum Disorder (ASD) in learning and social activities is vital but challenging. In this work, engagement refers to sustained attention, active involvement and appropriate responsiveness during interaction, which are important for social, cognitive and learning skill development (Kishida and Kemp, 2009; Salam et al., 2017; Sidner et al., 2005). However, many autistic children experience challenges in sustaining attention, responding to social cues, and participating in learning activities (Baron-Cohen, 1995; McWilliam and BaileyJr, 1995). As a result, interaction with peers, parents, teachers, and therapists plays a crucial role in supporting their developmental progress (Keen, 2009).

Social robots are becoming valuable tools in autism interventions because they can provide predictable, repetitive, and motivating interactions, which help maintain children’s attention (Lalmas et al., 2014; Ricks et al., 2010; Scassellati et al., 2012). Reliable engagement recognition is important for Robot-Assisted Autism Therapy (RAAT) because it enables robots to adapt their behavior. Drejing et al. (2015) further expanded on engagement, defining it through observable behaviors and their intensity. Recent studies have explored engagement recognition in children with ASD during robot interactions. Al-Nafjan et al. (2023) used facial feature detection with Convolutional Neural Network (CNN) models but noted challenges with subtle reactions. Rudovic et al. (Rudovic et al., 2018) demonstrated that deep learning models can effectively estimate engagement intensity from facial images collected during robot-assisted autism therapy. Kim et al. (2023) proposed a deep learning framework for joint engagement classification that leverages RGB video and skeleton-based representations, demonstrating improved performance through video augmentation and highlighting its potential for real-time human–robot interaction, while Vaufreydaz et al. (2016) demonstrated improved reliability by integrating multimodal data. Despite these advances, these methods are difficult to compare directly because they are evaluated on different cohorts, feature representations, and data splits, and several limitations persist in the field: i) few publicly available datasets containing data of children with ASD; ii) small cohorts and task-specific recordings; iii) evaluation protocols dominated by random splits, under which per-child and per-session information leaks between the training and testing sets and inflates reported accuracy.

To address these limitations, this paper introduces QAMQOR, a benchmark dataset collected during real RAAT sessions with 34 children with ASD. The dataset contains frame-level engagement annotations and heterogeneous visual representations: face, body and hand pose features. All features were extracted from ≈47 hours of video data collected across 187 therapy sessions. This scale supports realistic, deployment-oriented evaluation of engagement recognition in RAAT, while remaining representative of the data volumes achievable in clinical settings, where recruiting children with ASD is inherently constrained. The main contributions of this work are: i) introducing QAMQOR, a publicly available dataset for engagement recognition during RAAT; ii) defining four evaluation protocols based on Random, Child-Independent, Session-Independent, and Activity-Independent splits for realistic assessment of model generalization; iii) providing reproducible baseline results for binary and multi-class engagement classification using representative classical, temporal and sequence-learning models, including a recent Temporal Convolutional Network (TCN), each evaluated under the same windows, protocols and untuned hyperparameters so that models are compared under matched rather than individually optimized conditions; and iv) demonstrating empirically that the choice of evaluation protocol affects reported performance to a degree comparable to, and for fine-grained engagement classification approximately twice as large as, the choice of classifier among discriminative models, which underscores the need to evaluate engagement recognition under realistic deployment scenarios. Rather than introducing a novel classification algorithm, this work contributes a reproducible benchmarking framework for engagement recognition under standardized, leakage-controlled evaluation protocols, enabling fair comparison of future methods on equal terms.

## 2 Related work

### 2.1 Engagement recognition in RAAT

Social robots have increasingly been adopted in therapeutic interventions for children with ASD. RAAT interventions have shown promise in improving children’s communication skills by eliciting responses and creating a safe, engaging environment that can be more appealing than traditional therapeutic tools (Andriella et al., 2018; Bharatharaj et al., 2017; Clabaugh et al., 2019; Conti et al., 2020; Sandygulova et al., 2019). Research suggests that robots can adapt to autistic children’s unique needs, fostering positive mood changes through their human-like appearances and embedded communication models (Andrist et al., 2015; Costa et al., 2014; van den Ber et al., 2020). However, personalizing humanoid robots to use multimodal communication and social cues effectively remains a significant challenge in human-robot interaction (HRI). Most current systems still rely on limited annotated datasets and on human assessment procedures by therapists or researchers, which are expensive, subjective, and difficult to scale (Al-Nafjan et al., 2023; Kim et al., 2012; Kotsi et al., 2025; Rudovic et al., 2018).

Engagement assessment in RAAT often involves analyzing social cues, such as eye gaze, hand gestures, facial expressions, body posture, and tone of voice (Yu et al., 2010). Given that children with ASD may struggle to display these behaviors, multimodal approaches are essential for assessing and supporting their engagement (Scassellati et al., 2012). Recent approaches include gaze tracking, head movement analysis, and machine learning models that predict engagement labels, all of which contribute to tailoring therapy to individual children’s needs (Castellano et al., 2009; Jain et al., 2020; Javed et al., 2020; Lemaignan et al., 2016).

More recent engagement-recognition systems have moved toward deep and temporal architectures. Abedi et al. (2021) combined a residual network with a TCN to model engagement as a spatio-temporal problem on the DAiSEE dataset. Lin et al. (2026) employed three-dimensional CNNs to jointly recognise behavioral and emotional engagement from short classroom video segments, while Das and Dev (2025) extracted OpenFace and MediaPipe features and systematically compared classical and deep models, reporting that gradient-boosted trees such as XGBoost remained the strongest classifiers. Although these methods advance the state-of-the-art in the broader educational domain, they are trained and evaluated on heterogeneous cohorts, sensing modalities and label schemes. Their reported accuracies are therefore not directly comparable, and none addresses RAAT under Child-Independent evaluation. This absence of a common, leakage-controlled benchmark motivates the resource introduced in this work.

### 2.2 Datasets involving children with ASD and social robots

As interest in socially assistive robots continues to grow, the scarcity of high-quality public datasets remains a major obstacle to progress in this field. Due to privacy concerns, most HRI datasets are not publicly accessible, especially those related to child-robot interaction (CRI). Table 1 summarizes representative datasets involving children, autism intervention, and social robots, highlighting differences in cohort size, sensing modalities, and application scope.

**TABLE 1:** Comparison of publicly reported HRI datasets relevant to child-robot interaction and autism intervention research.

Dataset	Year	Participants	Application context	Data modalities	Availability	Predefined evaluation splits
Keepon Pro-Active Dataset (Kozima et al., 2009)	2005–2009	> 30 children (2–4 years)	Child–robot interaction, ASD observations	Video, audio, longitudinal interaction sessions	Restricted	None released
MDCA Dataset (Rudovic et al., 2017)	2017	35 children with ASD (aged 3–13 years)	NAO-based autism therapy, affect and engagement analysis	Video, audio, behavioral labels, engagement, valence, arousal	Upon request	None released
DE-ENIGMA Dataset (Riva and Riva, 2020; Schadenberg and Evers, 2023)	2020	128 children with ASD	Robot-assisted emotion learning and social imagination training	RGB video, audio, depth, facial expressions, body gestures, affect annotations	Restricted	None released
DREAM Dataset (Billing et al., 2020)	2020	61 children with ASD (aged 3–6 years)	Robot-enhanced vs. human-enhanced therapy	3D gaze, head pose, body motion, metadata	Upon request	None released
Engagnition Dataset (Kim et al., 2024)	2024	57 children with ASD (4–16 years)	Serious-game intervention for ASD engagement recognition	GSR, skin temperature, accelerometer, gaze, intervention logs, usability/workload questionnaires, 3-level engagement labels, video/audio	Public	None released
QAMQOR (proposed)	2026	34 children with ASD (3–12 years)	Robot-assisted autism therapy engagement benchmarking	Video-derived face, body, hand, full-frame features, frame-level engagement labels	Public	Random, Child-Independent, Session-Independent, Activity-Independent

The influence of evaluation design and data imbalance on reported performance has only recently begun to receive systematic attention. Whitehill et al. (2014) evaluated engagement classifiers under subject-independent cross-validation and cautioned against selecting hyperparameters on the test set, using nested cross-validation to avoid optimistic bias, while Lin et al. (2026) adopted a video-level split after observing that treating clips from the same recordings as independent samples risks data leakage. Class imbalance compounds this problem: Abedi et al. (2021) reported that, without correction, engagement classifiers collapse toward the majority class, and that weighted-loss and balanced-sampling strategies recover minority classes only at the expense of overall accuracy. Recent surveys of affective computing identify limited dataset scale, class imbalance, and generalization across users as persistent open challenges, underscoring the need for balanced benchmark datasets and standardized evaluation protocols (Yazici et al., 2026). These leakage- and imbalance-aware practices, however, remain study-specific, and no publicly available resource systematically evaluates generalization to previously unseen children, sessions and activities in RAAT.

The DREAM dataset (Billing et al., 2020) collected multimodal recordings from 61 children with autism during two therapy conditions: with a robot (RET, n=30 ) and with humans (SHT, n=31 ) using three high-resolution RGB cameras and two Kinect (RGB-D) cameras. The Multimodal Dataset of Children with Autism (MDCA), introduced by Rudovic et al. (2017), included 35 children from two countries and focused on affective and task engagement during NAO-mediated therapy. Another notable effort is the DE-ENIGMA dataset (Riva and Riva, 2020; Schadenberg and Evers, 2023), which contains more than 150 h of multimodal recordings collected from 128 children with ASD during robot-assisted and adult-assisted emotion learning activities. The dataset includes audio, video, depth recordings, facial expressions, body posture cues and affect-related annotations. However, DE-ENIGMA primarily focuses on emotion recognition, affect analysis and social imagination training rather than engagement recognition. More recently, Engagnition (Kim et al., 2024) provided a publicly available multimodal CRI dataset involving 57 participants for engagement analysis collected during a serious-game intervention. Other relevant resources include the PInSoRo dataset (Lemaignan et al., 2018), which studied free-play CRI, and long-term in-home socially assistive robot datasets targeting disengagement prediction (Jain et al., 2020).

Despite these valuable contributions, several important challenges remain for engagement recognition in RAAT. Existing datasets differ substantially in sensing modalities, interaction settings, annotation protocols and accessibility, making direct comparison between approaches difficult. Furthermore, few publicly available datasets provide standardized evaluation protocols that systematically assess model generalization to previously unseen children, therapy sessions and activity types.

To address these limitations, we introduce QAMQOR, a benchmark dataset specifically designed for engagement recognition in RAAT. Unlike the Keepon Pro-Active dataset (Kozima et al., 2009), which primarily documents early CRI studies, DREAM (Billing et al., 2020), which compares robot-enhanced and human-enhanced therapy, MDCA (Rudovic et al., 2017), which focuses on affective and task-engagement analysis, DE-ENIGMA (Schadenberg and Evers, 2023), which targets emotion learning and social imagination training, and Engagnition (Kim et al., 2024), which investigates engagement during serious-game interaction, QAMQOR provides a standardized benchmark for engagement recognition during real RAAT sessions. In addition, QAMQOR defines four complementary evaluation protocols: Random, Child-Independent, Session-Independent and Activity-Independent splits. These enable systematic assessment of model generalization under realistic deployment conditions. By evaluating performance across unseen children, sessions and activities, QAMQOR facilitates fair, reproducible and clinically relevant benchmarking of engagement recognition methods.

Table 1 shows that although several CRI datasets are now accessible, none are distributed with predefined, leakage-controlled evaluation splits. QAMQOR is positioned not as the largest such resource, but, to the best of our knowledge as the first among the RAAT/CRI dataset to release predefined leakage-controlled evaluation protocols alongside the data.

## 3 Dataset collection

We collected data during RAAT sessions involving children with ASD, therapists, and the humanoid NAO robot. Following the therapeutic methodology designed for ASD therapy (Sandygulova et al., 2019), sessions included structured activities designed to promote imitation, joint attention, turn-taking and communication behaviors (Zhanatkyzy et al., 2023).

The therapy protocol included several activity groups. “Dances” encouraged active movement and emotional wellbeing through music-based interaction. “Songs” combined music with simple choreography recommended by a music therapist to promote listening, rhythm, and coordinated movements. “Storytelling” involved the robot acting out short fairy tales with gestures, sounds, and character behavior to sustain attention and emotional engagement. “Touch Me” asked children to identify robot body parts by touching tactile sensors in response to spoken prompts. “Imitations” targeted motor imitation skills through themed actions. “Emotions” aimed to develop affect recognition and joint attention, with the robot demonstrating emotional expressions linked to pictures and situational examples. “Social Acts” encouraged imitation of everyday non-verbal communication (handshaking, hugging, clapping, etc.) behaviors.

The robot delivered spoken instructions, demonstrated actions, provided auditory cues, and expressed praise after responses. The therapist provided additional guidance and behavioral support when required. Each session lasted approximately 10–20 min, depending on the child’s level of engagement and attention span. Each session comprised 3–6 short activities selected by the therapist according to the child’s responsiveness. Activities were launched manually through the control interface. All sessions were recorded using a wide-angle camera for later annotation and behavioral analysis.

This study received ethical approval from the University and the Republican Children’s Rehabilitation Center (RCRC). At RCRC, specialists conducted diagnostic tests and therapists observed the children on their first day. Parents or caregivers gave their informed written consent to participate in this study.

### 3.1 Participants

The study involved 34 children previously diagnosed with ASD by qualified clinicians according to standard clinical procedures. The participants were aged 3–12 years (mean =5.7 , SD =2.24 ). Figure 1 presents the age distribution of participants. The cohort included 32 boys and 2 girls, reflecting the higher reported prevalence of ASD in males.

**FIGURE 1:**
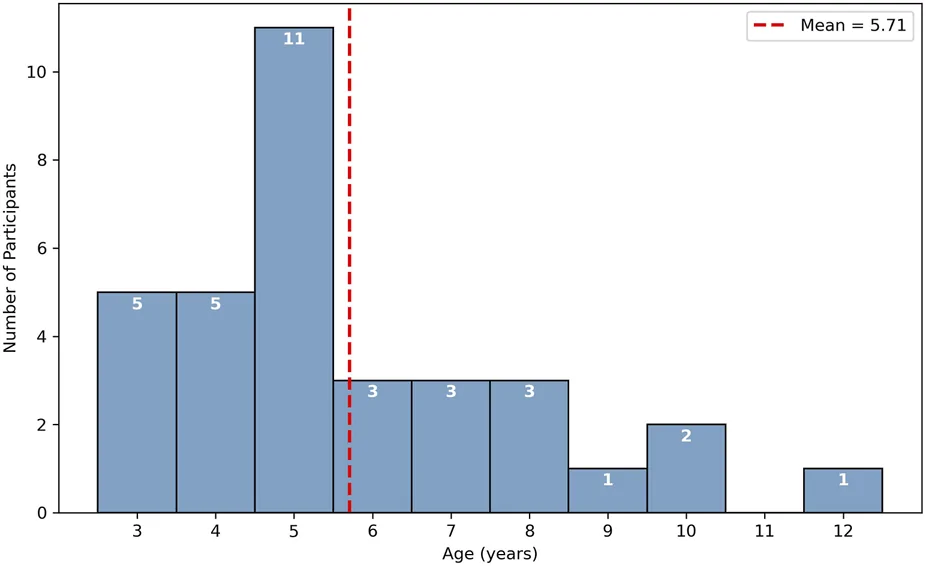
Age distribution of participants included in the QAMQOR dataset.

ADOS-2 assessments were available for all participants at the RCRC. ADOS-2 is a standardized assessment of autism-related communication, social interaction and behavior (Lord et al., 2012). A therapist with 7 years of experience completed the assessment. ADOS-2 severity scores range from 1 to 10, where higher scores indicate greater ASD symptom severity (1 – lowest level; 2−3 - mild; 4−5 - moderate; 6−7 - moderately high; and 8−10 - high to very high severity) (Hus et al., 2014). The reported average score was 6.53 (SD =1.74 ). This indicates moderate-to-high ASD symptom severity across the cohorts. As ADOS-2 module selection depends on language ability, age, and developmental profile, these scores are presented descriptively rather than as a pooled diagnostic threshold for all participants. Twenty-three children were non-verbal, while eleven were verbal. Co-occurring ADHD was reported for 12 participants. For descriptive analysis, participants were grouped into three developmental age groups: 3–4 years (n=10) , 5–6 years (n=13) , and 7–12 years (n=11) . This grouping was used to summarize the characteristics and participation in the session rather than for predictive modeling.

Table 2 provides the demographic characteristics of the children and the recording statistics of the collected therapy sessions. The verbal status and ADHD categories reported in Table 2 are not mutually exclusive. Of the 12 children with co-occurring ADHD, 3 were verbal and 9 were non-verbal.

**TABLE 2:** Participant characteristics and session recording statistics for the QAMQOR dataset.

Characteristic	Value
Number of children	34
Age, mean ± SD (range), years	5.7±2.24 (3–12)
Sex, n (male/female)	32/2
Verbal status, n (verbal/non-verbal)	11/23
Co-occurring ADHD, n	12
ADOS-2 severity score (mean ± SD)	6.53 ± 1.74
Sessions and recordings	
Total therapy sessions, n	187
Total recording duration	≈ 47 h
Annotation sampling rate	1 fps

### 3.2 Dataset

Datasets are fundamental for machine learning applications (Saleh, 2019). We developed a visual feature extraction pipeline to organize the RAAT study data for training and evaluating machine learning models. The pipeline consists of six steps (Figure 2):

- Extracting frames from each video using the OpenCV library video capture function (Bradski, 2000), at a rate of 1 fps.
- Detecting humans and cropping frames accordingly (Redmon and Farhadi, 2018).
- Removing unnecessary images, leaving only the frames that contain the child.
- Assigning engagement labels to each frame: multi-class (1–5) and binary (0 and 1) classification (Figure 3).
- Parsing the cropped frames using the OpenPose library (Cao et al., 2019) and the MediaPipe Holistic library (Lugaresi et al., 2019), then saving the extracted keypoints in a JavaScript Object Notation (JSON) file format.
- Generating the dataset by extracting facial features and body joints from the JSON files into a single CSV file.

**FIGURE 2:**
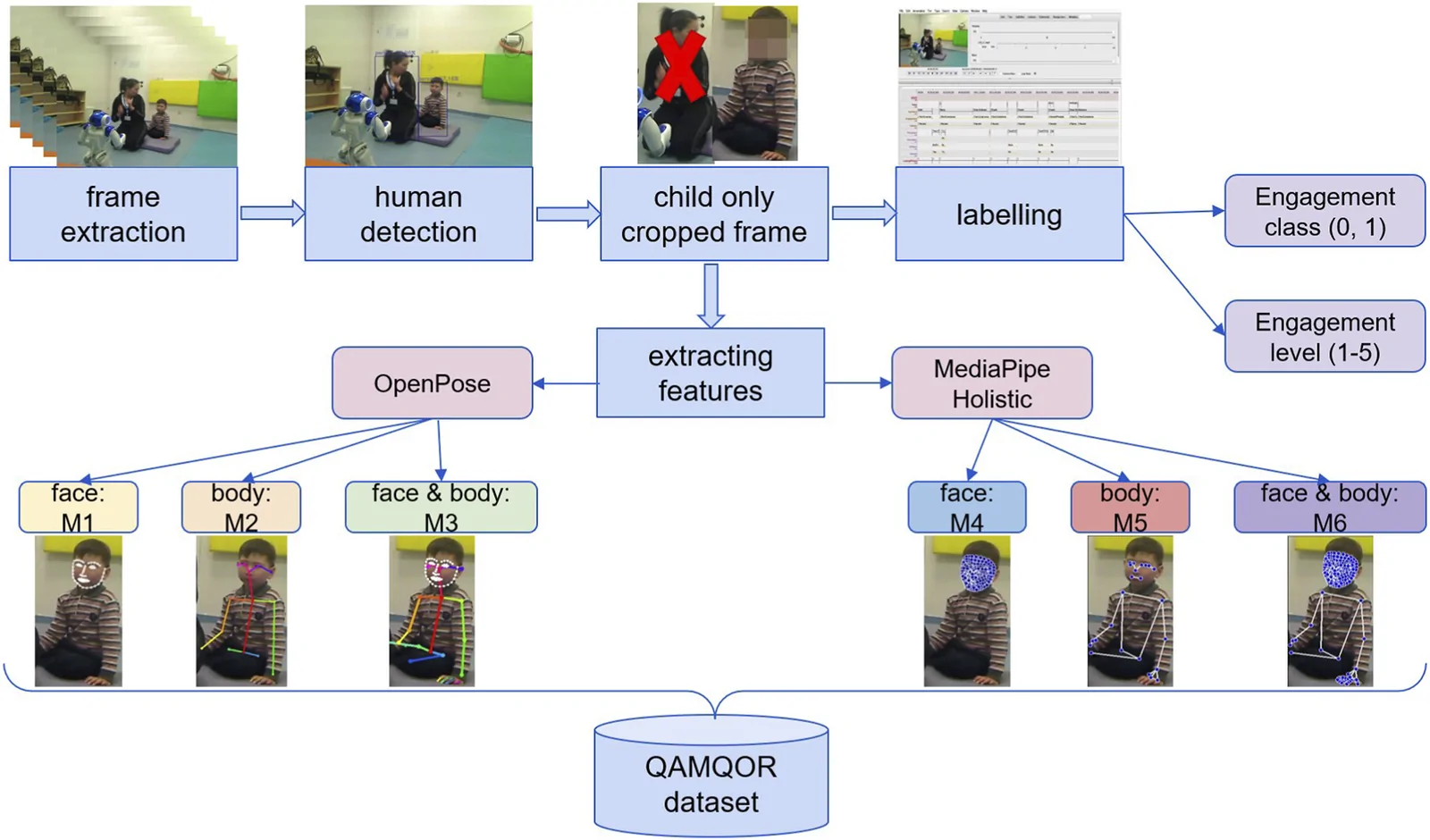
Overview of the data pre-processing steps. Raw therapy videos were sampled at 1 fps, processed to detect and crop children, annotated with engagement labels and extracted features using OpenPose and MediaPipe Holistic libraries. The resulting feature representations were organized into six modalities: M1 (OpenPose face keypoints), M2 (OpenPose body keypoints), M3 (OpenPose face and body keypoints), M4 (MediaPipe face keypoints), M5 (MediaPipe body and hand keypoints), M6 (MediaPipe face, body and hand keypoints).

**FIGURE 3:**
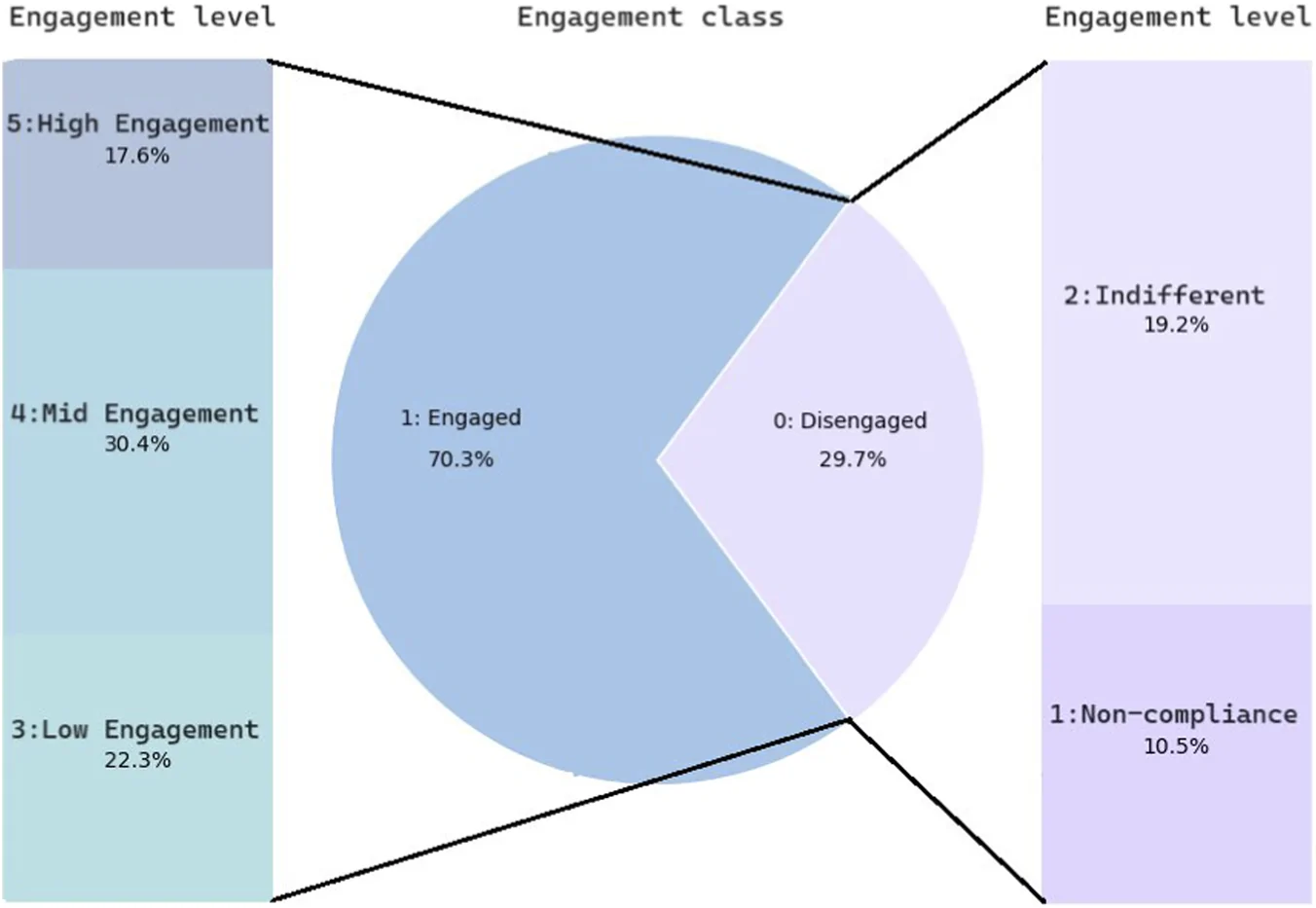
Frequency distribution of engagement labels in the QAMQOR dataset.

Frames were sampled at a rate of 1 fps to align with the annotation protocols. Engagement labels were assigned at 1-s intervals. Unlike action recognition tasks that often require high frame rates to capture rapid motion patterns, engagement recognition focuses on behavioral states that generally persist over longer time intervals. Therefore, a higher frame sampling rate would produce multiple visually similar frames that are associated with the same engagement label. In addition, consecutive video frames in RAAT sessions often exhibit substantial redundancy, as engagement levels typically evolve over several seconds rather than within individual frames. The use of 1 fps sampling reduced storage and computational requirements while preserving the temporal granularity required for frame-level engagement annotation and benchmarking.

This pipeline transformed the raw recordings into a structured dataset suitable for machine learning.

### 3.3 Coding

Engagement is an important metric in autism therapy. It is defined by active and appropriate involvement in the task with the robot and/or the therapist. To annotate the dataset, we adapted the Likert-type coding framework (0–5) used in previous studies (Kim et al., 2012; Rudovic et al., 2018) to ensure comparability with other researchers. Because scores of 0 and 1 both represented strong disengagement, these categories were merged. Consequently, a five-level scale was used:

- Engagement label 1 (non-compliance), when a child is unwilling to engage in the conversation, is not paying attention to the engagement, and is not reacting to the therapist;
- Engagement label 2 (indifferent), if the therapist asks the same question more than three times before the child follows the rules;
- Engagement label 3 (low engagement), after two or three repetitions, the child complies with the directions;
- Engagement label 4 (mid engagement), when a child follows directions the first time but needs assistance, such as a finger point, a name call, a demonstration of something to pay attention to, etc.;
- Engagement label 5 (high engagement), when the child spontaneously interacts with the NAO robot, responds to the therapist and/or robot, and completes the necessary tasks.

Unlike previous studies that coded fixed intervals or task segments (Kim et al., 2012; Rudovic et al., 2018), labels were assigned at 1-s intervals to capture rapid changes in behavior commonly observed in children with autism.

Three annotators performed the coding. Prior to annotation, all annotators completed a training session based on a written coding manual containing behavioral definitions and worked examples. To quantify inter-rater reliability, the three annotators independently coded a common 20% subset of the recordings, sampled to span multiple children, sessions and activity types. Agreement on this subset was computed on the five-level engagement scale using a two-way random-effects, absolute-agreement intraclass correlation coefficient (ICC(2,1), yielding ICC =0.826 , which indicates good agreement among raters, based on the interpretation proposed by Koo et al. (Koo and Li, 2016). For the shared subset, final labels were resolved by majority agreement. The remaining recordings were then partitioned among the three annotators and coded independently.

Figure 3 illustrates the natural class imbalance in the engagement labels. This imbalance reflects the natural distribution of children’s engagement during therapy sessions, where moderate and high engagement levels occur more frequently than strong disengagement. This original distribution was retained for benchmarking rather than altered through resampling. The rationale and the corresponding imbalance-aware evaluation metrics are detailed in Section 4.2.

### 3.4 Feature extraction tools

Reliable feature extraction is essential for automatic engagement recognition. Children’s behavioral responses are often expressed through facial expressions, gaze direction, body posture, and hand movements.

For keypoint-based feature extraction, we employed the OpenPose and MediaPipe Holistic libraries. From each video frame of children with ASD, OpenPose was used to extract 70 face and 25 body keypoints, while MediaPipe Holistic was used to extract 468 face, 33 pose, and 42 hand keypoints. A Gaussian blur filter was applied to each frame to reduce noise and improve feature quality.

OpenPose successfully extracted 88.2% of facial keypoints, 77.3% of left-hand keypoints, 68.2% of right-hand keypoints, and 100% of body keypoints. MediaPipe Holistic achieved comparable performance, with successful extraction rates of 87.6% for facial keypoints, 74.7% for both left- and right-hand keypoints, and 100% for body keypoints. Overall, OpenPose showed slightly better performance for facial and left-hand detection, whereas MediaPipe Holistic provided more reliable right-hand detection. Using two independent tools enabled comparison of detection performance while reducing the impact of missing landmarks.

## 4 Experimental methodology

We evaluated the QAMQOR dataset using four widely adopted classifiers (LightGBM, CatBoost, XGBoost and Logistic Regression), a Temporal XGBoost model, a Long Short-Term Memory (LSTM) network and a TCN (Figure 4). LightGBM, CatBoost, XGBoost and Logistic Regression predict from a single frame and are referred to throughout as the static frame-level models. Temporal XGBoost is referred to as the temporal model, and the LSTM and the TCN as the sequence-learning models. To evaluate whether the conclusions derived from conventional machine learning baselines remain valid for temporal models, we additionally included representative temporal and two sequence-learning baselines. Temporal XGBoost represents lightweight handcrafted temporal feature aggregation, and the LSTM and the more recent TCN represent end-to-end sequence learning. The classifiers establish reproducible frame-based baselines, and the temporal and sequence-learning baselines let us test whether these protocol effects also hold once short-term dynamics are modeled. Together, these approaches enable comparison of static, feature-engineered temporal and sequence-learning models under identical evaluation protocols.

**FIGURE 4:**
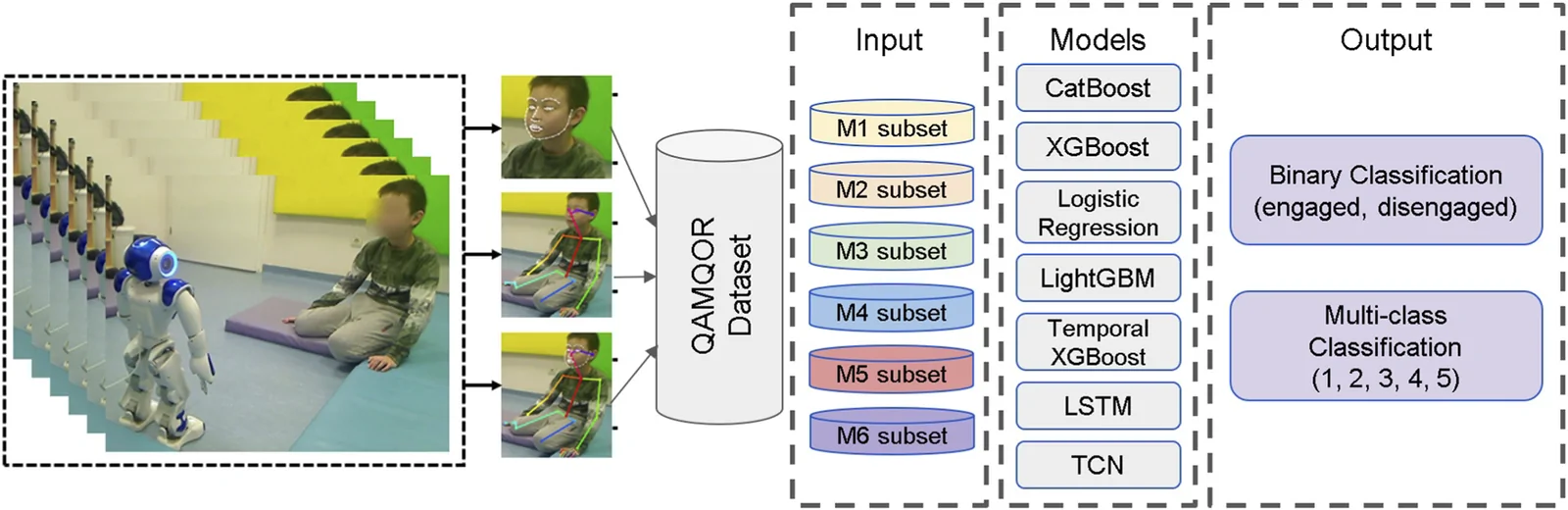
Overview of the engagement recognition architecture used to evaluate the QAMQOR dataset.

LSTM was selected as a representative sequence-learning baseline for comparison with conventional machine learning methods, rather than to establish the optimal deep learning architecture for engagement recognition. LSTM is one of the most widely adopted recurrent neural network architectures for modeling temporal dependencies in sequential data (Donahue et al., 2017). We report its behavior without modification to provide a transparent benchmark for future sequence-learning approaches. The tendency of both sequence baselines toward majority-class prediction is characterized in Section 5.2.

All conventional machine learning models were implemented in Python using Scikit-learn, XGBoost, LightGBM and CatBoost, whereas the LSTM and TCN models were implemented using TensorFlow/Keras. Before training, missing values resulting from unsuccessful keypoint extraction were replaced with a constant value of zero, denoting an undetected keypoint. Because this constant imputation does not depend on any data statistics, it is applied identically across all partitions and introduces no information leakage between the training and test sets. Features were standardized when required by the learning algorithm, with standardization parameters estimated on the training partition only and applied unchanged to the test partition. All models, including Temporal XGBoost, the LSTM and the TCN, were trained and evaluated using the same splits and benchmark protocols to ensure a fair comparison.

### 4.1 Evaluation protocols

Four evaluation protocols were defined using different splits. For all experiments, 80% of the data were allocated for training and 20% for testing. A fixed random seed of 42 was used for all randomized operations.

In the Random split, the samples were randomly divided into training and testing sets. This split yields an optimistic reference point, because partitioning ignores child and therapy session identity; near-identical frames from a single recording can populate both training and testing sets.

In the Child-Independent split, data from unseen participants were used for testing. This split reflects a realistic deployment scenario in which the model must generalize to previously unseen children.

In the Session-Independent split, whole therapy sessions were held out for testing. Sessions are nested within children, so a child contributing several sessions may appear in both sets. This split assesses robustness to previously unseen therapy sessions while minimizing subject overlap.

In the Activity-Independent split, training and testing were separated according to therapeutic activity type. This evaluation protocol assesses model generalization across different therapeutic activities.

All splits were generated once using a fixed random seed to define a stable benchmark configuration, such that future methods can be compared against identical splits. We acknowledge that a single partition cannot capture performance variability across alternative splits.

### 4.2 Prediction tasks and metrics

We considered two prediction tasks: i) binary engagement and ii) multi-class engagement classification.

As illustrated in Figure 3, the engagement labels are imbalanced, with higher engagement levels occurring more frequently than lower engagement levels. Therefore, for a multi-class task, macro-average precision, recall and F1-score were reported to weigh all engagement levels equally regardless of their frequency. For the binary task, macro-averaging was likewise adopted so that both engaged and disengaged classes contributed equally to the reported F1-score, avoiding the optimistic bias introduced by positive-class or frequency-weighted F1 under the 70.3% majority class. Consequently, F1-score denotes macro-F1 throughout this paper and is reported alongside balanced accuracy (macro-averaged recall) and Cohen’s κ (a chance-corrected agreement coefficient). Accuracy is reported for consistency with previous work, but is interpreted alongside the majority-class baseline, as a classifier that always predicts the dominant class can achieve high accuracy while providing little discriminative power. The main benchmark applies no class re-weighting, over-sampling or under-sampling because the main objective of QAMQOR is to establish reproducible benchmark results using the original clinical data distribution. Preserving the natural class frequencies enables future engagement recognition methods to be evaluated under the same clinically realistic conditions. To additionally assess whether imbalance handling alters the relative standing of the models, we report an imbalance-aware variant of the models in a separate Section 4.6.

Hyperparameters were selected manually through preliminary experiments on the training data and then fixed across the four benchmark evaluation protocols to ensure a fair comparison among all evaluated models. This follows recommended practice for leakage-free evaluation, in which hyperparameters are not selected on the test set (Whitehill et al., 2014). Extensive hyperparameter optimization was intentionally omitted because the objective of QAMQOR is to establish reproducible baseline performance rather than maximize classifier accuracy. Table 3 summarizes the hyperparameter settings used in all experimental evaluations.

**TABLE 3:** Hyperparameters used in the benchmark experiments.

Model	Hyperparameters
Logistic Regression	max_iter = 3000
LightGBM	n_estimators = 300, learning_rate = 0.03, random_state = 42, verbose = −1
CatBoost	iterations = 300, learning_rate = 0.03, verbose = 0
XGBoost	n_estimators = 300, learning_rate = 0.03, max_depth = 8, subsample = 0.8, colsample_bytree = 0.8, eval_metric = logloss (binary)/mlogloss (multi-class), random_state = 42
Temporal XGBoost	num_boost_round = 300, eta = 0.03, max_depth = 8, tree_method = hist, max_bin = 256, objective = logistic (binary)/softprob (multi-class), random_state = 42, window = 10; window descriptor = current frame ⊕ windowed mean ⊕ windowed standard deviation ⊕ first-order frame difference
LSTM	LSTM units = 64, dense units = 32, dropout = 0.3, 0.2, optimizer = Adam, batch size = 32, epochs = 100, validation split = 0.2, early stopping (patience = 10), ReduceLROnPlateau (factor = 0.5, patience = 5), window = 10
TCN	3 residual blocks, filters = 64, kernel size = 3, dilations = 1,2,4, dropout = 0.2, global average pooling, dense units = 32, optimizer = Adam, batch size = 256, epochs = 100, early stopping (patience = 10), ReduceLROnPlateau (factor = 0.5, patience = 5), window = 10

By publicly releasing the evaluation scripts, evaluation protocols, engagement annotations, and extracted OpenPose and MediaPipe keypoints, QAMQOR enables reproducible evaluation and direct comparison of future engagement recognition methods under consistent experimental conditions.

### 4.3 Temporal XGBoost evaluation

Temporal modeling is important because engagement evolves through pose transitions, gaze shifts, repetitive movements, and CRI dynamics. To investigate whether temporal feature engineering improves standard machine learning performance, we implemented a lightweight Temporal XGBoost framework using keypoints extracted with OpenPose and MediaPipe Holistic.

Consecutive frames were grouped using a sliding window of 10 frames. Each window was transformed into a fixed-length descriptor comprising the current keypoint vector, temporal mean, temporal standard deviation and first-order frame difference features (Bulling et al., 2014; Lara and Labrador, 2013). These descriptors were then classified using the XGBoost algorithm.

The Temporal XGBoost serves as a lightweight temporal baseline that captures short-term behavioral trends while remaining compatible with tree-based classifiers. Because temporal information is represented through aggregated statistics rather than explicit sequence modeling, it does not preserve the full temporal ordering of behavioral events or model long-range behavioral dependencies.

### 4.4 Deep sequence-learning baselines

We additionally evaluated the QAMQOR dataset using the LSTM network. In contrast to Temporal XGBoost, which summarizes temporal information using statistical aggregation, the LSTM directly learns temporal dependencies from sequential pose features through recurrent memory cells.

For each sample, consecutive keypoint vectors were grouped into temporal windows of 10 frames. Each sequence was used as the input to the LSTM network. For a fair comparison of the models, the same window size was adopted for Temporal XGBoost, the LSTM and the TCN.

The LSTM architecture consisted of a single recurrent layer followed by two fully connected layers with dropout regularization. Model-specific hyperparameters are shown in Table 3.

Binary engagement recognition employed a sigmoid activation with binary cross-entropy loss, and multi-class recognition used a softmax activation with sparse categorical cross-entropy loss. Training employed early stopping and adaptive learning-rate scheduling to reduce overfitting.

We also evaluated a TCN as a more recent architecture. It captures patterns over time using stacked dilated causal convolutions with residual connections, rather than recurrence. The network has three residual blocks, each with 64 filters and a kernel size of 3. The dilation rates ( 1,2 and 4) let the receptive field cover the full 10-frame window. This is followed by global average pooling and the same output layer used in the LSTM. The TCN uses the same window size, optimizer, early-stopping schedule and random seed as the other sequence model. Hyperparameters were not tuned per protocol for any model, so that all architectures are compared under the same conditions rather than under individually optimized ones. This design supports statements about relative performance under a common, untuned configuration. It does not support statements about the maximum attainable performance of any architecture, and the sequence-model results are accordingly interpreted as a lower bound throughout.

### 4.5 Statistical analysis

All statistical comparisons were performed on macro-F1 unless otherwise stated. Differences among classifiers and evaluation protocols were assessed using the Friedman test with Kendall’s W as the effect size. Classifier comparisons were conducted over matched evaluation settings (same modality, splits, and task), whereas evaluation protocols were compared separately for the binary and multi-class tasks using each classifier-modality combination as a matched block. Significant Friedman tests were followed by Wilcoxon signed-rank *post hoc* tests with Holm–Bonferroni correction. Kendall’s W measures how consistently models are ranked across matched blocks, not how far apart they are. Therefore, we also report the range of mean macro-F1 across the levels of each factor. A factor can produce a consistent ranking (large W ) while separating models by only a small margin. For the classifier factor, we report this range over all seven models and over the five models that learned a discriminative mapping (the four static frame-level models and Temporal XGBoost), since the two deep sequence baselines collapsed to majority-class prediction in some settings and inflated the apparent spread.

### 4.6 Imbalance-aware variants

The engagement labels are strongly imbalanced (Figure 3). Because of this, a model that always predicts the majority class already reaches ≈70% accuracy on the binary task and 30% on the multi-class task. To test whether handling this imbalance affects the performance of the models, we ran balanced versions of the models under the same conditions.

For these balanced versions, we made two changes. First, we standardized features using statistics from the training set only. Second, we applied balanced (inverse-frequency) class weights during training, so that rarer classes carried more weight. For the deep models, we also applied these weights to the validation set. This way, early stopping was guided by balanced performance rather than by majority-class accuracy.

These settings were the same across all protocols and modalities, and were never chosen based on the test set. Balancing is meant to improve the imbalance-sensitive metrics, not accuracy. We therefore judge these variants mainly by macro-F1, balanced accuracy and Cohen’s κ .

The balanced CatBoost and Temporal XGBoost variants exceeded the memory available on our hardware for the widest feature sets. Therefore, they are reported on the subset of modalities where they ran, with the paired unbalanced values computed on the same subset. The five models with full coverage provide a consistent basis for the statistical comparison.

## 5 Results

### 5.1 Overall benchmark performance

The benchmark comprises 336 single-partition evaluations spanning seven classifiers, six visual modalities, four evaluation protocols, and two prediction tasks. In addition, XGBoost was evaluated under 5-fold Child-Independent cross-validation (CV) across the six modalities and two tasks (Table 4).

**TABLE 4:** Overall benchmark performance of all evaluated models.

Model	Accuracy (%)	Macro-F1	Balanced accuracy (%)	Cohen’s κ
Binary classification task				
CatBoost	72.5±3.3	0.47±0.04	52.0±2.0	0.05±0.05
LightGBM	72.9±4.0	0.50±0.06	53.7±3.6	0.10±0.09
Logistic Regression	71.9±3.4	0.47±0.04	51.9±2.0	0.05±0.05
Temporal XGBoost	72.9±4.5	0.53±0.06	55.3±4.0	0.13±0.10
XGBoost	73.3±4.8	0.53±0.08	55.1±5.4	0.13±0.13
XGBoost (Child-Independent CV)	71.8±0.4	0.48±0.03	51.6±1.0	0.04±0.03
LSTM	72.1±2.9	0.45±0.05	51.2±2.3	0.03±0.06
TCN	72.1±2.8	0.45±0.05	51.3±2.1	0.03±0.05
Majority-class baseline	70.3	0.41	50.0	0.00
Multi-class classification task				
CatBoost	34.7±5.0	0.23±0.05	25.3±4.2	0.08±0.06
LightGBM	36.4±9.0	0.29±0.10	28.9±8.9	0.13±0.12
Logistic Regression	33.1±4.6	0.24±0.05	24.9±3.9	0.07±0.06
Temporal XGBoost	36.6±9.3	0.29±0.10	29.5±9.0	0.13±0.12
XGBoost	37.4±10.5	0.30±0.12	30.0±10.5	0.14±0.14
XGBoost (Child-Independent CV)	28.1±0.6	0.19±0.02	21.8±1.0	0.03±0.02
LSTM	32.2±3.6	0.15±0.05	21.4±2.5	0.02±0.04
TCN	32.7±3.3	0.14±0.05	21.0±1.9	0.02±0.03
Majority-class baseline	30.4	0.09	20.0	0.00

Values are reported as mean ± standard deviation across all evaluation settings. “XGBoost (Child-Independent CV)” denotes 5-fold Child-Independent cross-validation (CV), pooled across the six modalities.

Across all settings, binary engagement recognition reached a mean accuracy of 72.5% , but a mean macro-F1 of only 0.49, a mean balanced accuracy of 52.9% and a mean Cohen’s κ of 0.08. These values are close to the majority-class baseline (accuracy =70.3% , macro-F1 =0.41 , balanced accuracy =50.0% , κ=0.00 ), indicating that high binary accuracy is largely attributed to the dominant class rather than to learned engagement discrimination. Multi-class recognition was substantially harder, with a mean accuracy of 34.7% , macro-F1 of 0.23, balanced accuracy of 25.9% and κ of 0.09, compared with a majority-class baseline of 30.4% accuracy and macro-F1 =0.09 .

Among individual configurations, the strongest binary result was achieved by XGBoost (accuracy =80.6% , macro-F1 =0.69 ; OpenPose M2 , Random split), and the strongest multi-class result was also obtained by XGBoost (accuracy =58.2% , macro-F1 =0.55 ; OpenPose M2 , Random split). Both best cases occurred under the Random split. Figure 5 shows that binary results form a compact cluster whose macro-F1 (≈0.41−0.69) barely separates from the majority-class baseline. Multi-class results are far more dispersed (accuracy ≈24−58% , macro-F1 ≈0.10−0.55 ), reflecting the greater difficulty and stronger sensitivity to the evaluation protocol, modality and classifier.

**FIGURE 5:**
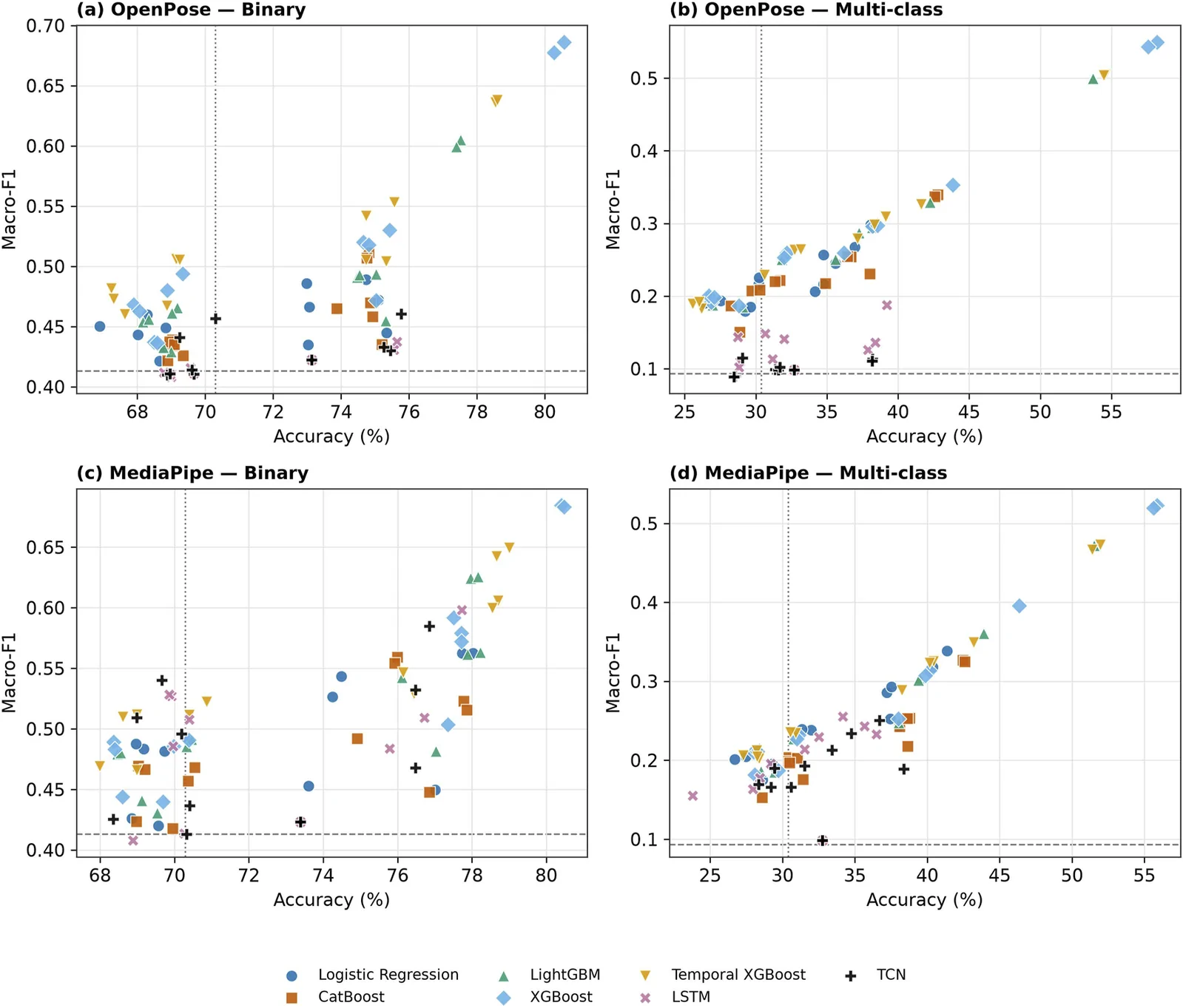
Relationship between accuracy and macro-F1 across all experimental evaluations (dashed lines: majority-class baseline). Results are presented separately for the feature extraction method and classification task: **(a)** OpenPose–binary classification; **(b)** OpenPose–multi-class classification; **(c)** MediaPipe–binary classification; **(d)** MediaPipe–multi-class classification.

Relative to this majority-class baseline, configurations under the Child-Independent and Session-Independent protocols exhibit balanced accuracy near 50.0% and κ close to zero, confirming that much of the apparent binary accuracy reflects the dominant class rather than genuine engagement discrimination. Both deep sequence models (LSTM and TCN) remained at this baseline level, suggesting that the near-baseline performance reflects the difficulty of the task under these evaluation protocols rather than the limitations of any single classifier. The role of class imbalance is examined in Section 5.6.

### 5.2 Effect of the classifier

A Friedman test over matched evaluation settings indicated a large and statistically significant effect of classifier choice on macro-F1. For the binary task, $\chi^{2}\left(6\right)=86.46,p<0.001$ , Kendall’s W=0.60 , and for the multi-class task, $\chi^{2}\left(6\right)=102.98$ , p<0.001 , W=0.72 . In contrast, the effect on accuracy was small and non-significant for both tasks (binary: $\chi^{2}\left(6\right)=10.16$ , p=0.12 , W=0.07 ; multi-class: $\chi^{2}\left(6\right)=11.19$ , p=0.08 , W=0.08 ). This divergence indicates that accuracy conceals classifier differences under class imbalance, whereas macro-F1 exposes them. The classifier effect was small in magnitude among models that learned a discriminative mapping. In the binary task, mean macro-F1 ranged from 0.47 (CatBoost) to 0.53 (Temporal XGBoost). In the multi-class task, it ranged from 0.23 (CatBoost) to 0.30 (XGBoost). Both ranges are about 0.07. Including the two deep sequence baselines widens them to 0.09 and 0.16. This widening reflects their collapse toward majority-class prediction, not a difference in architectural capacity. Post-hoc Wilcoxon signed-rank tests with Holm–Bonferroni correction confirmed that XGBoost and Temporal XGBoost were the two strongest classifiers, with no significant difference between them, and that XGBoost, Temporal XGBoost and LightGBM significantly outperformed Logistic Regression (adjusted p<0.05 ). Among these three top classifiers, LightGBM was significantly outperformed by both XGBoost and Temporal XGBoost (adjusted p<0.05 ). CatBoost did not follow the pattern of the other boosted trees. It ranked among the weakest classifiers and was significantly outperformed by XGBoost, Temporal XGBoost and LightGBM in both tasks, and did not differ significantly from Logistic Regression. The two deep sequence models ranked lowest overall. They did not differ significantly in the binary task. In the multi-class task the LSTM was significantly above the TCN (p=0.01) , but the difference of 0.02 macro-F1 is of no practical consequence, as both remained at the majority-class baseline. Because these baselines collapsed to majority-class prediction under the Random split (Table 4), this ordering should be interpreted as evidence of the difficulty of training deep sequence models on small clinical datasets with heterogeneous participants rather than as a fair comparison of architectural capacity.

On average across modalities and evaluation settings, XGBoost, Temporal XGBoost and LightGBM outperformed Logistic Regression in mean macro-F1 for both the binary and multi-class classification tasks (Figure 6), whereas CatBoost did not. The margin was nonetheless modest. The best-performing model exceeded Logistic Regression by ≈0.06 macro-F1 in both tasks (Temporal XGBoost in the binary task, XGBoost in the multi-class task), and LightGBM by 0.03 (binary), and 0.05 (multi-class). Therefore, gradient-boosted trees performed best among the evaluated models, but by a small margin. XGBoost achieved the strongest overall performance in the multi-class classification task. Among the static frame-level models it also achieved the highest accuracy under Random, Session-Independent and Activity-Independent protocols and was marginally higher under the Session-Independent protocol ( 30.5% , tied with LightGBM). In the binary task the accuracy ordering was less stable and depended on which models were compared. Among the static frame-level models, CatBoost was highest under the Session-Independent split (69.0%) , while LightGBM and Logistic Regression were effectively tied under the Activity-Independent protocol ( 76.3% for both). Neither ordering held across the full set of models. The LSTM reached the highest binary accuracy under the Session-Independent split (69.2%) and Temporal XGBoost under the Activity-Independent protocol (76.6%) . This instability is consistent with the non-significant Friedman result for accuracy reported above.

**FIGURE 6:**
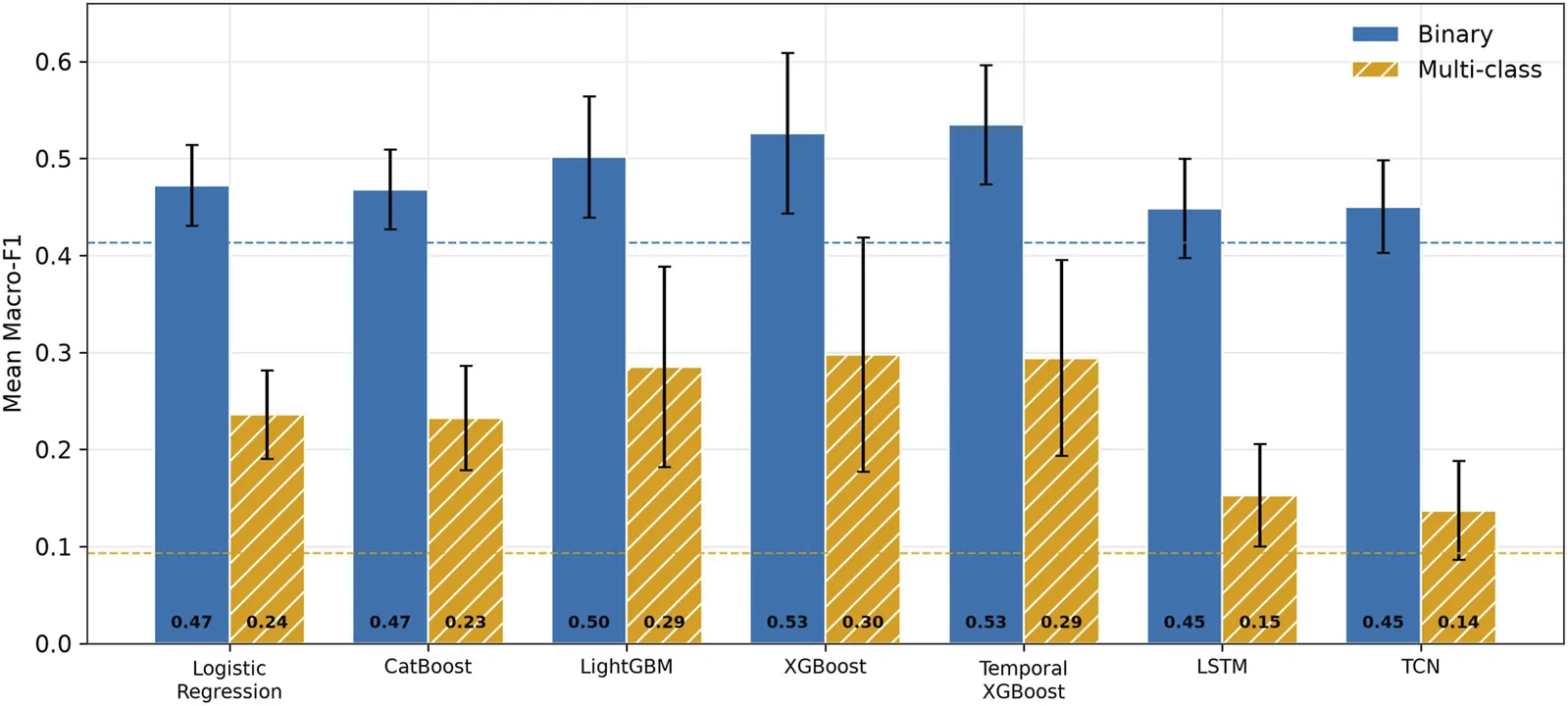
Average macro-F1 achieved by each classifier across all modalities and evaluation settings for binary and multi-class engagement recognition tasks (dashed lines: majority-class baseline).

In contrast, CatBoost was the weakest of the gradient-boosted ensembles across most settings. In the binary task under the Child-Independent split, its mean macro-F1 fell below that of Logistic Regression (0.44 vs. 0.45). Its binary performance was comparable to the untuned deep sequence baselines rather than to the other boosted-tree classifiers. This pattern indicates that the default CatBoost configuration was poorly suited to these high-dimensional keypoint features.

The deep sequence baselines LSTM and TCN did not improve on the boosted trees and, in several settings, converged toward majority-class prediction. As shown in Table 4, their balanced accuracy stays near chance (LSTM 51.2% binary, 21.4% multi-class; TCN 51.3% binary, 21.0% multi-class), with κ≈0.02−0.03 for both tasks. This collapse was complete under the Random split, where the networks defaulted to the dominant class across all six modalities (M1−M6) , producing macro-F1 at the majority-class baseline ( ≈0.42 binary and ≈0.10 multi-class) with κ near zero. We report these results without modification to document the difficulty of training deep sequence models on small, imbalanced clinical CRI data. The deep sequence-learning results should therefore be read as a lower bound on such models’ performance rather than as an optimal architecture.

#### 5.2.1 Temporal XGBoost vs. static XGBoost

Temporal aggregation produced small but systematic gains under the generalization protocols. As shown in Table 5 by comparing the two models on identical modalities, Temporal XGBoost slightly exceeded static XGBoost on the binary task for Child-Independent (+0.03) , Session-Independent (+0.02) and Activity-Independent (+0.03) splits and on the multi-class task for the Session-Independent (+0.02) and Activity-Independent (+0.01) splits. In both tasks, the static model retained an advantage only on the optimistic Random split ( −0.04 for both), where visually similar frames from the same child or session appear in both sets and reduce the value of temporal context. These findings suggest that short-term temporal statistics (mean, standard deviation and first-order differences over a 10-frame window) contribute information that is most useful precisely when the model must generalize beyond the training distribution, even though such aggregation cannot preserve the full temporal ordering of behavioral events. The strongest temporal results were 79.0% accuracy, macro-F1 =0.65 for the binary task (MediaPipe M6 , Random) and 54.6% accuracy, macro-F1 =0.50 for the multi-class task (OpenPose M2 , Random).

**TABLE 5:** Comparison between static XGBoost and Temporal XGBoost, reported as mean macro-F1 over the modalities common to both tasks. Positive gains indicate that temporal aggregation improved performance.

Task	Split	Static macro-F1	Temporal macro-F1	Gain	Better model
Binary	Child	0.46	0.49	+0.03	Temporal
Binary	Session	0.47	0.49	+0.02	Temporal
Binary	Activity	0.53	0.56	+0.03	Temporal
Binary	Random	0.64	0.60	−0.04	Static
Multi-class	Child	0.20	0.20	0.00	-
Multi-class	Session	0.22	0.24	+0.02	Temporal
Multi-class	Activity	0.29	0.30	+0.01	Temporal
Multi-class	Random	0.48	0.44	−0.04	Static

### 5.3 Effect of evaluation protocol

The evaluation protocol had a large and highly significant influence on macro-F1. For the binary task, the Friedman test revealed $\chi^{2}\left(3\right)=74.54,p<0.001$ , Kendall’s W=0.59 (large effect). Mean macro-F1 was highest under the Random split (0.52) followed by the Activity-Independent split (0.50), and lowest under the Session-Independent (0.46) and Child-Independent (0.45) splits (Figure 7); mean accuracy followed a similar ordering (Activity-Independent 76.3% , Random 75.5% , Child-Independent 69.4% and Session-Independent 68.9% ). Post-hoc Wilcoxon tests with Holm–Bonferroni correction confirmed that the Random split significantly outperformed both the Child-Independent and Session-Independent splits (both p<0.001 ). Similarly, the Activity-Independent split significantly outperformed both the Child-Independent and Session-Independent splits (p<0.001) .

**FIGURE 7:**
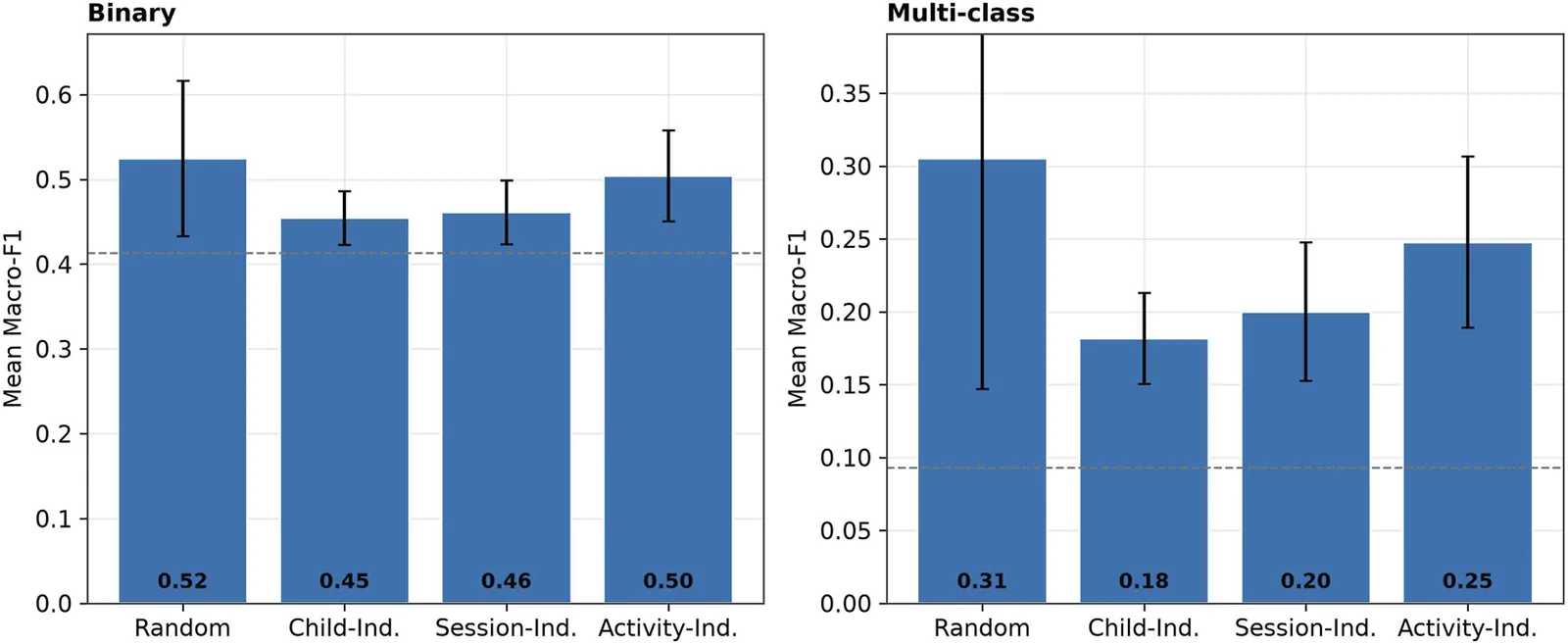
Mean macro-F1 by evaluation protocol obtained for binary and multi-class engagement recognition.

Multi-class classification remained protocol-sensitive, $\chi^{2}\left(3\right)=54.77,p<0.001$ , Kendall’s W=0.43 (moderate). Mean macro-F1 fell from 0.31 (Random) to 0.25 (Activity-Independent), 0.20 (Session-Independent) and 0.18 (Child-Independent), with mean accuracy dropping from 42.4% (Random) to 30.4% (Session-Independent) and 28.5% (Child-Independent). Post-hoc tests showed that the Random split significantly outperformed Child-Independent and Session-Independent splits (both p<0.001 ) and, more narrowly, the Activity-Independent split (p=0.003) . The Activity-Independent split significantly outperformed the Child-Independent and Session-Independent splits (both p<0.001 ). The Child-Independent and Session-Independent splits also differed significantly (p<0.001) by only 0.02 macro-F1. Under these two challenging protocols, macro-F1 approached the majority-class baseline (static classifier mean =0.20 for Child-Independent and =0.21 for Session-Independent, with κ near zero). Temporal XGBoost exhibited the same trend. OpenPose multi-class macro-F1 increased from 0.19 (Child-Independent) to 0.45 (Random) and MediaPipe binary macro-F1 rose from 0.50 to 0.61.

The protocol effect is at least as large as the classifier effect. In the multi-class task, mean macro-F1 varied by 0.12 across protocols but by only 0.07 across the five discriminative classifiers. Therefore, the protocol effect is about twice as large. In the binary task, the two effects were comparable (0.07 for both). Kendall’s W is larger for the classifier factor in the multi-class task ( W=0.72 vs. W=0.43 ). This reflects ranking consistency, not a larger effect on the metric scale.

These results confirm that random partitioning overstates deployable performance, because it allows frames from the same child or session to inform both model fitting and evaluation. The resulting inflation is as large as the entire spread between the weakest and strongest discriminative classifiers, and larger still for fine-grained engagement classification. Therefore, reliable benchmarking requires reporting performance under realistic generalization protocols, not under random splits alone.

### 5.4 Effect of visual modalities

Modality choice affected performance in a task-dependent manner (Figure 8).

**FIGURE 8:**
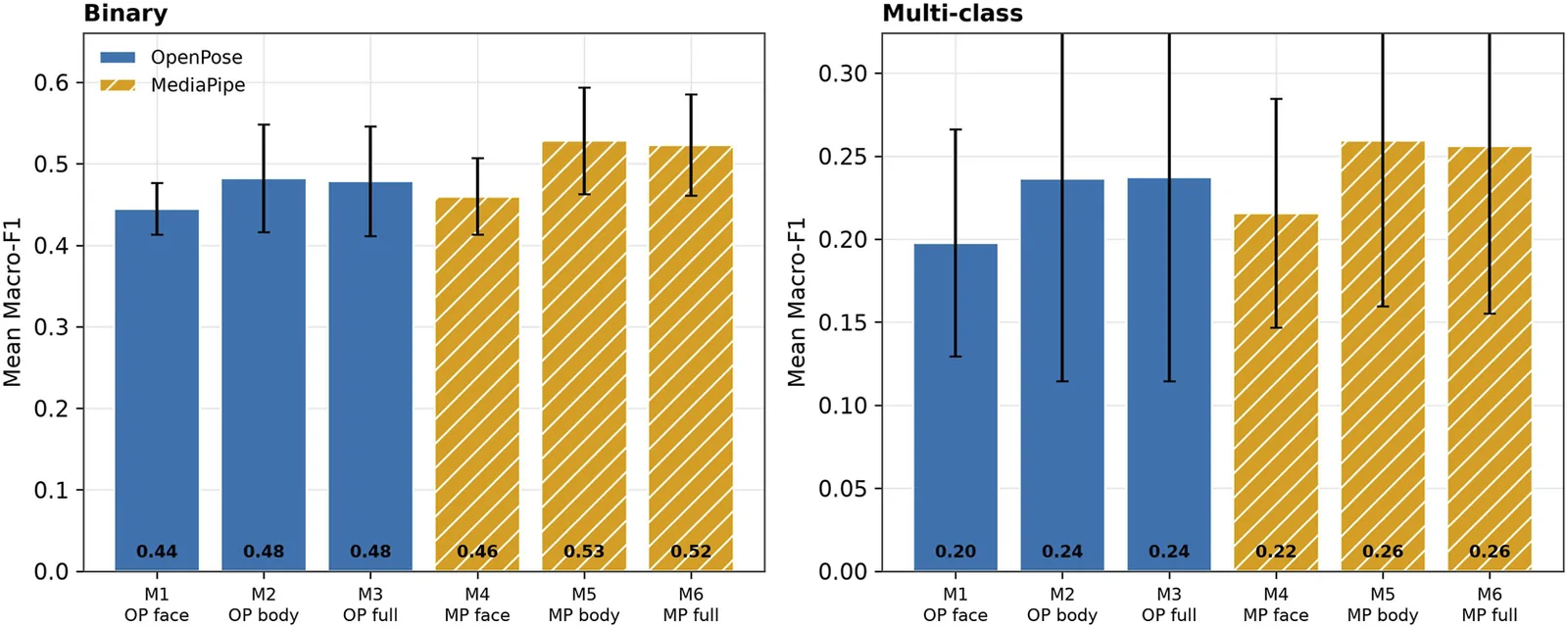
Mean macro-F1 by visual modalities (blue: OpenPose, orange: MediaPipe) across all evaluation protocols.

For the multi-class task, modalities containing body keypoints were the most informative. MediaPipe body and full representations ( M5 and M6 ) reached macro-F1 =0.26 , marginally ahead of the corresponding OpenPose representations ( M2 and M3 , macro-F1 =0.24 ). The two face modalities ( M1 , macro-F1 =0.20 ; M4 , macro-F1 =0.22 ) were the weakest. This suggests that posture, movement initiation, imitation responses, and gross-motor responses are stronger indicators of therapeutic engagement than facial cues alone.

For the binary task, the pattern shifted towards modalities extracted using MediaPipe ( M5 macro-F1 =0.53 and M6 macro-F1 =0.52 ), followed by OpenPose modalities ( M2 and M3 , macro-F1 =0.48 ). As in the multi-class task, the two face modalities ( M1 , macro-F1 =0.44 ; M4 , macro-F1 =0.46 ) were among the weakest.

M1 modality, which contains face keypoints extracted using the OpenPose library, was competitive in binary classification but weaker for multi-class classification. Facial features may help distinguish engaged and disengaged states, but they appear insufficient to capture subtle differences between low, moderate, and high engagement.

The combined representations M3 (OpenPose face and body) and M6 (MediaPipe face, body and hand) often improved robustness under the Session-Independent and Child-Independent splits, but did not consistently outperform smaller modality subsets, as the redundancy and noise introduced by higher-dimensional inputs can offset the benefit of additional keypoints. This suggests that targeted behavioral representations may be more effective than simply maximizing the number of keypoints.

### 5.5 Evaluation of tools

A task-dependent difference was observed between the two extraction tools (Figure 9). MediaPipe Holistic features dominated in binary recognition (mean macro-F1 0.50 vs. 0.47; mean κ 0.10 vs. 0.05). On the multi-class task this advantage nearly disappeared (mean macro-F1 0.24 vs. 0.22; mean κ 0.09 vs. 0.08; mean accuracy 34.9% vs. 34.6% ). This small multi-class advantage of MediaPipe is attributable to these deep sequence baselines (LSTM and TCN), whose near-majority-class predictions lowered the OpenPose average more than the MediaPipe average. Once these deep sequence baselines are excluded, the two tools are indistinguishable on the multi-class task (mean macro-F1 0.27 for both), indicating that OpenPose and MediaPipe body keypoints carry comparable information for fine-grained engagement recognition.

**FIGURE 9:**
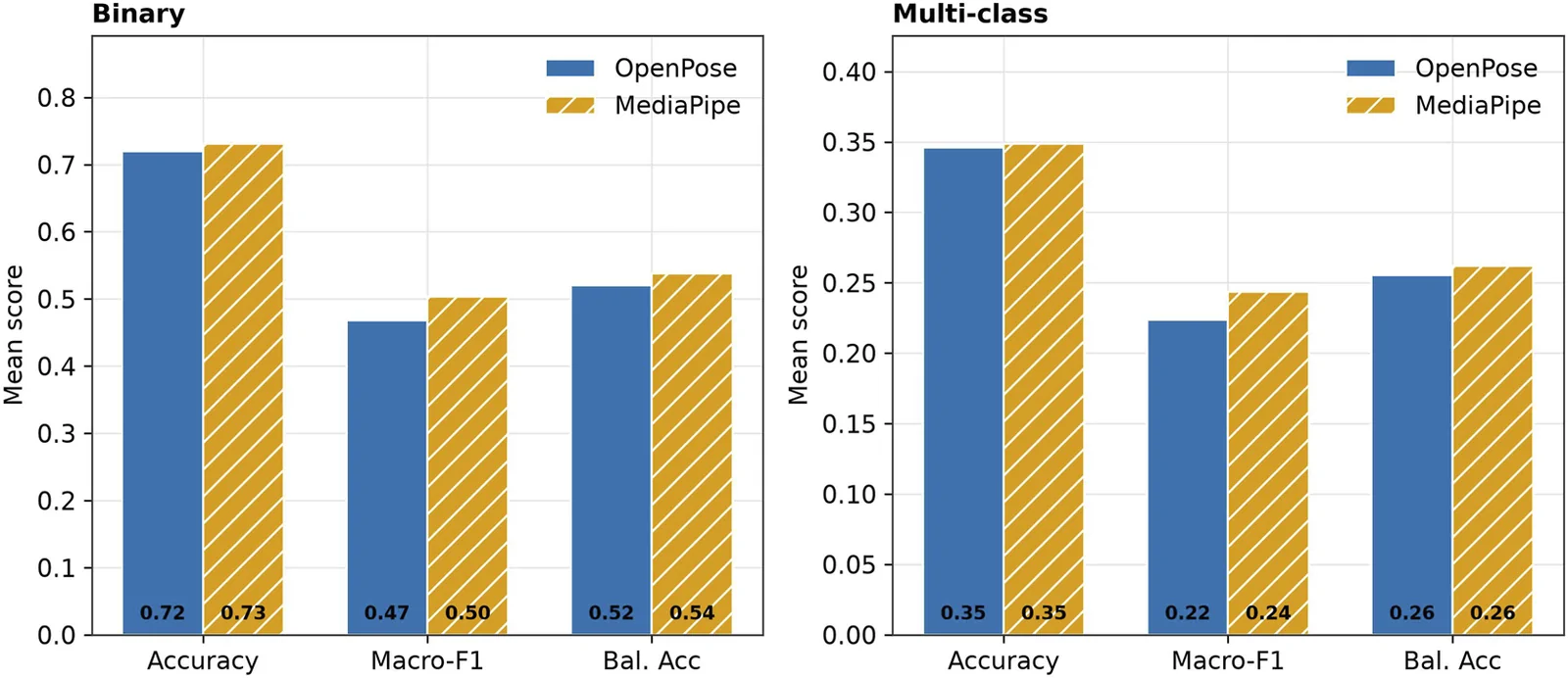
Comparison of engagement recognition performance using OpenPose and MediaPipe feature extraction tools.

Overall, MediaPipe provided the more robust keypoints for the binary task, whereas for fine-grained multi-class recognition the two tools carried comparable information once the deep sequence baseline s were set aside.

### 5.6 Effect of class balancing

The preceding sections show that near-baseline behavior in high-capacity models can distort aggregate comparisons. This raises the question of whether imbalance handling would change the relative standing of the models. Therefore, we evaluated an imbalance-aware variant of each model and compared it against its unbalanced counterpart (Table 6). The effect was consistent across every model family. Class balancing improved the imbalance-sensitive metrics: macro-F1, balanced accuracy and Cohen’s κ . At the same time, it lowered raw accuracy. This is because the balanced models no longer default to the majority class. As detailed in Table 6, Cohen’s κ increased for every model on both tasks, while overall accuracy decreased. The direction of this effect was identical for the linear, tree and deep models, reinforcing that accuracy alone is misleading on this dataset, since a majority-class predictor already attains approximately 70% (binary) and 30% (multi-class) accuracy.

**TABLE 6:** Effect of class balancing on each model, reported as the mean of each metric across evaluation settings (unbalanced → balanced).

Model	Accuracy (%)	Macro-F1	Balanced accuracy (%)	Cohen’s κ
Binary classification task				
XGBoost	73.3→65.7	0.53→0.60	55.1→60.9	0.13→0.20
LightGBM	72.9→62.1	0.50→0.58	53.7→60.2	0.10→0.18
Logistic Regression	71.9→58.4	0.47→0.55	51.9→58.0	0.05→0.13
LSTM	72.1→52.9	0.45→0.50	51.2→54.5	0.03→0.07
TCN	72.1→56.5	0.45→0.51	51.3→55.1	0.03→0.08
CatBoost	71.9→59.6	0.45→0.56	51.3→58.5	0.03→0.14
Temporal XGBoost	72.2→67.1	0.52→0.59	54.5→60.0	0.12→0.19
Multi-class classification task				
XGBoost	37.4→33.6	0.30→0.31	30.0→33.2	0.14→0.15
LightGBM	36.4→31.6	0.29→0.30	28.9→32.2	0.13→0.14
Logistic Regression	33.1→26.8	0.24→0.25	24.9→27.7	0.07→0.09
LSTM	32.2→22.1	0.15→0.20	21.4→24.4	0.02→0.04
TCN	32.7→24.4	0.14→0.19	21.0→23.9	0.02→0.04
CatBoost	34.5→28.4	0.23→0.26	25.2→29.4	0.08→0.10
Temporal XGBoost	36.6→34.5	0.30→0.32	29.6→32.4	0.14→0.15

Two findings relate directly to the comparison with more recent architectures. First, giving every model the same imbalance handling did not change the outcome: the deep sequence baselines still do not surpass the simpler balanced baselines. A Friedman test over the five balanced models with full modality coverage (XGBoost, LightGBM, Logistic Regression, LSTM and TCN) showed a significant effect of classifier choice on macro-F1 (binary: $\chi^{2}\left(4\right)=44.27,p<0.001$ , Kendall’s W=0.46 ; multi-class: $\chi^{2}\left(4\right)=50.30,p<0.001$ , Kendall’s W=0.52 ). Balanced XGBoost and LightGBM ranked highest, and the balanced LSTM and TCN ranked lowest. On the Child-Independent, Session-Independent and Activity-Independent protocols, which matter most for deployment, the balanced tree models reached the highest accuracy and κ , with the balanced deep models clustered slightly below. Second, even after balancing, both deep models remained at chance under the Random split, confirming that their strong-looking Random split behavior reflects the class prior rather than transferable engagement signals. Class balancing improved every model on the imbalance-aware metrics, but it did not change how the models ranked. This rules out the absence of class weighting as an explanation for the near-baseline behavior of the sequence models. Three candidate explanations remain: the number of independent participants, the behavioral heterogeneity of the cohort, and the coarse temporal resolution. Under matched, untuned conditions, a more recent temporal architecture did not outperform the simpler baselines on QAMQOR.

## 6 Discussion

Accurate engagement recognition is crucial for adaptive RAAT, since robots must interpret the child’s behavior in order to personalize interaction. This study introduced QAMQOR, a publicly available dataset for engagement recognition in RAAT and established a standardized evaluation framework to assess model generalization. Unlike existing datasets, which typically report performance on a single train-test set, QAMQOR defines four benchmark evaluation protocols (Random, Child-Independent, Session-Independent and Activity-Independent). These allow us to assess model robustness under realistic deployment conditions.

The main finding is that the evaluation protocol influences reported performance at least as much as classifier selection. The classifier effect was statistically strong and consistent in rank order, but modest in magnitude once the two deep sequence baselines are set aside. It separated the weakest from the strongest discriminative model by 0.07 macro-F1 in both tasks. The protocol effect was comparable in the binary task (0.07) and about twice as large in the multi-class task (0.12). Classifier choice is reliably detectable but has limited practical consequence. Protocol choice can shift the reported result by more than the full span of the classifier comparison. High performance under the Random split should therefore not be read as evidence of generalization. It mixes each child’s and each session’s frames across both sets and is best interpreted as a leakage-inflated upper bound. Releasing standardized protocols is a prerequisite for fair, cumulative progress.

The Child-Independent split was among the hardest protocols. Engagement is expressed through child-specific behavioral cues, such as gaze, gross-motor responses and hand movements that differ across children. This variability is amplified by the clinical heterogeneity of ASD across a wide developmental range (ages 3–12). The mapping from pose features to engagement is therefore non-stationary across individuals. The gap between Random and Child-Independent splits also quantifies the potential value of per-child calibration, motivating personalization in RAAT.

Both deep sequence baselines, the LSTM and more recent TCN, collapsed to majority-class prediction under the Random split across six modalities (κ=0) , recovering only weak discrimination under the generalization protocols. The TCN behaved identically to the LSTM under the same configuration. Therefore, the behavior is not specific to one architecture. Neither model was tuned, so this shows only that added temporal capacity gave no advantage under these matched conditions. It does not show that such capacity is unusable here. Possible explanations are the small number of independent participants, the class imbalance, the 1 fps sampling and the behavioral heterogeneity of the cohort. The balancing experiment rules out class imbalance as the sole cause. Separating the remaining explanations would require larger cohorts or per-architecture tuning, both of which are left to future work. In contrast, Temporal XGBoost yielded small but consistent improvements under the Child-Independent, Session-Independent and Activity-Independent protocols, with no advantage on the Random split. Engagement therefore exhibits a short-term temporal structure that individual frames cannot capture, and that is most valuable when the model must generalize.

A natural objection is that deep models underperformed simply because they were not given class weights. Applying the same imbalance handling to every model tested this objection directly. Balancing improved all models on the imbalance-aware metrics, but it preserved their ordering. The deep sequence models remained at or below the balanced tree ensembles. Therefore, the absence of class weighting does not account for this near-baseline behavior.

### 6.1 Practical implications

The gap between the Random and Child-Independent protocols has a clear practical meaning. Performance measured without subject separation overstates what a deployed system would achieve with a new child. Therefore, benchmark results should be read as an upper bound, not as an expected operating point. The near-chance Child-Independent performance also means that a keypoint-based classifier is not yet reliable enough to drive fully autonomous adaptation for an unseen child. It is better positioned as an assistive signal that supports therapist judgment. The computational cost also matters, since RAAT is frequently deployed on constrained on-robot hardware. LightGBM reached macro-F1 0.50 (binary) and 0.29 (multi-class) at a median training time of 26 s (measured on CPU without GPU acceleration). This is approximately one-seventh the time required by the TCN (186 s under the same conditions), which reached only 0.45 and 0.14. These observations motivate the following recommendations:

1. The Child-Independent split should be a required reporting standard. It is the closest proxy for encountering a new child. Its near-chance performance shows that current classifiers are not deployment-ready without per-child calibration. Therefore, human-in-the-loop use, personalization and cross-child domain adaptation are priorities for translational work.
2. Performance should be reported with imbalance-aware metrics. Accuracy near the majority baseline coincided with macro-F1 and κ near chance. Therefore, accuracy alone can mask a complete absence of engagement discrimination.
3. Results from the Random split should not be reported in isolation. Activity-Independent evaluation is not an adequate substitute: in the binary task, the two did not differ significantly (p=0.09) . A Random split should be accompanied by a Child-Independent or Session-Independent result obtained under the same configuration. Leakage is then quantified rather than assumed. The protocols released with QAMQOR allow future methods to be compared on an equal footing.
4. Lightweight models remain preferable in this data regime. They matched or exceeded the deep-sequence models on every metric at a lower cost. We report training rather than inference time, so this concerns on-site retraining and calibration. A full deployment assessment would also require per-frame latency and memory footprint.

### 6.2 Limitations

Several limitations should be considered. QAMQOR includes fewer participants than several existing datasets (Table 1), although its ≈47 h of continuously annotated recordings yield a comparable frame-level sample size. The cohort therefore remains small for training modern deep learning models. Both deep sequence baselines (LSTM and TCN) collapsed toward majority-class prediction, which suggests that the binding constraint is the number of independent participants rather than the number of annotated frames. Because recruiting children with ASD for clinical recording is inherently constrained, larger multi-site cohorts are the most direct route to improving generalization. The cohort was also markedly skewed toward male participants (32 boys, 2 girls), reflecting the higher reported prevalence of ASD in males. This limits sex-specific analyses and may constrain generalizability across sexes. The data were collected at a single rehabilitation center with one robot platform and therapy protocol, so cross-site and cross-platform generalization remains to be established. QAMQOR also does not include a typically developing control group. The dataset was designed specifically to support engagement recognition in children with ASD attending RAAT.

The pipeline relies exclusively on keypoint-based features and does not incorporate audio, prosody or physiological signals. In addition, the 1 fps sampling rate limits temporal resolution and may fail to capture fast behavioral cues. A further limitation of the temporal baselines is that they aggregate statistics rather than model explicit sequences, and do not preserve the full temporal ordering of behavioral events.

Three limitations of the benchmark itself should also be noted. First, results are reported for single fixed splits, so only the cross-validated XGBoost rows carry a variance estimate. Extending cross-validation to every model would allow confidence intervals to accompany each reference result. Second, the deep sequence baselines were not tuned per protocol, so their near-baseline behavior should be read as a lower bound rather than as evidence about architectural capacity. Claims in this paper concerning these models are correspondingly restricted to matched, untuned conditions, and establishing their attainable performance would require a per-architecture search on the training sets. Third, the benchmark compares models only against each other. Published engagement recognition results are obtained on different cohorts, label schemes and evaluation protocols. Therefore, they are not directly comparable with ours. Establishing external reference points would require applying the QAMQOR protocols to a second public dataset.

### 6.3 Future work

Future work follows two directions. The first concerns the data. Larger multi-site cohorts with more balanced recruitment would improve representativeness. Adding matched typically developing controls would enable comparative developmental analyses. Extending the recordings to audio, prosody and physiological signals would allow multimodal fusion. Recent surveys catalog fusion strategies in detail (Yazici et al., 2026). The second concerns the models. Transformers and graph neural networks over skeleton keypoints may capture temporal structure that statistical aggregation cannot. Self-supervised and transfer learning offer a route to exploiting limited labeled data. Cross-child domain adaptation and uncertainty-aware learning address the failure identified here, namely, the drop in performance on unseen children.

## Data Availability

The datasets presented in this study can be found in online repositories. The names of the repository/repositories and accession number(s) can be found below: https://github.com/nazerkerakhymbayeva/QAMQOR.
